# Antioxidant Properties of Ester Derivatives of Cinnamic and Hydroxycinnamic Acids in *Nigella sativa* and Extra-Virgin Olive Oils-Based Emulsions

**DOI:** 10.3390/antiox11020194

**Published:** 2022-01-20

**Authors:** Diego Romano Perinelli, Elisabetta Torregiani, Giulia Bonacucina, Marco Cespi, Giovanni Filippo Palmieri, Rosita Gabbianelli

**Affiliations:** 1 Chemistry Interdisciplinary Project (CHIP), School of Pharmacy, Via Madonna delle Carceri, University of Camerino, 62032 Camerino, Italy; diego.perinelli@unicam.it (D.R.P.); elisabetta.torregiani@unicam.it (E.T.); giulia.bonacucina@unicam.it (G.B.); marco.cespi@unicam.it (M.C.); gianfilippo.palmieri@unicam.it (G.F.P.); 2 Unit of Molecular Biology and Nutrigenomics, School of Pharmacy, University of Camerino, 62032 Camerino, Italy

**Keywords:** polyphenols, ferulic acid, cinnamic acid, *Nigella sativa* oils, extra-virgin olive oil (EVO)

## Abstract

New hydrophobic derivatives of cinnamic and hydroxycinnamic (ferulic and cumaric) acids obtained by chemical esterification of the carboxylic group with C10 linear alcohol were studied to evaluate their antioxidant capacity toward the superoxide anion and hydrogen peroxide in physiological buffer and in extra-virgin olive oil (EVO) or *Nigella sativa* oils. Results showed that cumaric and ferulic acids have higher antioxidants activity against superoxide anion and hydrogen peroxide than the other compounds. Cumaric acid and its C10-ester derivative were selected to be incorporated into EVO or *Nigella sativa* oil-based emulsions. The prepared emulsions had a comparable particle size distribution (in the range of 3–4 µm) and physical stability at least up to three months. *Nigella sativa* oil-based emulsions loaded with cumaric acid or its C10-ester showed a higher capacity to scavenger superoxide anion and hydrogen peroxide than EVO oil-based emulsions. In conclusion, cumaric acid or its C10-ester could promote the antioxidant properties of *Nigella sativa* oil when formulated as emulsions.

## 1. Introduction

Cinnamic and hydroxycinnamic (e.g., ferulic, cumaric, and caffeic) acids are naturally occurring chemical compounds belonging to the large family of bioactive polyphenols. These compounds are widely distributed in plants, and they represent one-third of phenol-derivatives assumed by humans with the diet [1]. In the last decades, cinnamic and hydroxycinnamic acids have received increasing interest in different fields, including nutraceutics, cosmetics, and biomedicine, since they are potent antioxidant compounds [2,3]. Thanks to their chemical structure, capable of scavenging free radicals, hydroxycinnamic acids behave as chain-breaking antioxidants. Specifically, the scavenging activity is related to the ability of the phenol group (-OH) to donate a hydrogen atom or an electron (transfer reaction mechanisms), resulting in the formation of a stable phenoxyl radical, in which the unpaired valence electron can be delocalized by resonance [4,5]. Structural modifications, mainly aromatic ring substitutions, have been made to improve the antioxidant activity [6,7]. However, the predominant hydrophilic nature of these compounds strongly limits their incorporation in fatty or oily materials, thereby limiting their use in hydrophobic matrices and membranes [1,2]. To overcome this issue, other structure modification strategies can be applied, such as the elongation of the conjugate lateral chain or modification of the carboxylic function such as esterification or amidation [8,9]. Extra-virgin olive (EVO) oil from *Olea europea* L. and *Nigella sativa* oil are functional foods and ingredients with well-documented beneficial effects on human health [10,11]. EVO oil contains a high amount of monounsaturated fatty acids (i.d., oleic acid) and low content of polyunsaturated fatty acids (i.d., linoleic and linolenic acids). Minor constituents are phenolic compounds and α-tocopherol [12,13]. *Nigella Sativa* oil contains a large variety of phytochemicals such as terpenes and terpenoids (limonene, carvone, α-thujene), phenolic compounds (trans-anethole, *p*-cymene, carvacrol), quinones (thymoquinone, thymohydroquinone, dithymoquinone), sterols, fatty acids, and alkaloids [14]. In particular, *Nigella* oil is highly rich in tymoquinone (30–48%), which has potent antioxidant and anti-inflammatory activities that account for the majority of the beneficial and therapeutic effects of this natural oil on human health [14,15]. As such, EVO and *Nigella* as pure oils were previously studied in cellular or animal models to assess their antioxidant and anti-inflammatory properties [16,17,18,19]. In order to facilitate the administration of natural oils both topically (e.g., across skin or mucosa) and systemically (e.g., oral route) and promote their absorption, the preparation of emulsified systems is a common strategy, which has been already widely applied for the vehiculation of EVO and *Nigella* oils with promising outcomes [20,21]. Thus, the present work is aimed at investigating the scavenging activities toward superoxide radical and hydrogen peroxide and the ability to inhibit hydroperoxide formation of new hydrophobic derivatives of cinnamic and hydroxycinnamic (ferulic and cumaric) acids obtained by chemical esterification of the carboxylic group with C10 linear alcohol. The antioxidant tests were performed in a physiological buffer, in EVO and *Nigella* pure oils, and in their emulsions stabilized using polysorbate 80 as an emulsifier. The ultimate goal was to assess whether these new phenolic derivatives can enhance the antioxidant potency of EVO and *Nigella* oils, especially in the case they are employed or administered to humans as emulsions.

## 2. Materials and Methods

### 2.1. Materials

Trans-cinnamic acid (purity ≥99%), *p*-cumaric acid (purity ≥98%), trans-ferulic acid (purity ≥99%), 1-decanol (purity 99%), *p*-toluenesulfonic acid, anhydrous toluene, luminol, H_2_O_2_, Tris-HCl buffer, lucigenin, xanthine, and xanthine oxidase were purchased from Sigma-Aldrich (St. Louis, Missouri, USA). DPPP was purchased from Cayman Chemical Co., Ann Arbor, MI, USA. Ethyl acetate, cyclohexane, ethanol, and potassium hydroxide (KOH) were purchased from Carlo Erba (Cornaredo, MI, Italy). Polysorbate 80 and phenolphthalein were purchased from A.C.E.F. (Fiorenzuola d’Arda, PC, Italy). *Nigella oil* from black cumin (*Nigella sativa* L.) seeds was produced by Tre Ponti Snr company (Polverigi, AN, Italy). Extra-virgin olive (EVO) oil from the Raggia cultivar of olive drupes (*Olea europaea* L.) was produced in a local olive mill. Chemical characterization of the EVO oil and *Nigella* oils has been reported in previous works [16].

### 2.2. Chemical Synthesis of Cinnammic, Cumaric, and Ferulic C10-Ester Derivatives

C10-ester derivatives of cinnamic, cumaric, and ferulic acid were synthesized according to the reported procedure [22]. Briefly, 2 g of cumaric acid, ferulic acid, or cinnamic acid were dispersed in anhydrous toluene (40 mL), then, 1-decanol was added (molar ratio 1:1.1) followed by p-toluenesulfonic acid (molar ratio 1:1.2), and the reaction mixture was refluxed for 4 h using a Dean–Stark apparatus (Thomas Scientific, Swedesboro, NJ, USA). Subsequently, the organic solvent was removed under vacuum, and *p*-toluenesulfonic acid was extracted using Na_2_CO_3_ from the crude product dissolved in ethyl acetate. The crude products were purified through a chromatographic column by using a cyclohexane/ethyl acetate 80:20 mixture as eluent. The final compounds were oils, and their chemical structures were confirmed by ^1^H-NMR spectroscopy (Varian EM-400 MHz spectrometer, Palo Alto, CA, USA).

### 2.3. Emulsion Preparation and Characterization

A total of 5% (*w/w*) of Nigella oil or EVO oil was added dropwise to a 1% (*w*/*w*) of polysorbate 80 solution under high-speed stirring (Ultraturrax T25 basic, IKA*^®^* Werke GmbH & Co. KG, Staufen, Germany) and homogenized at 9500 rpm for 5 min at room temperature. For loaded emulsions, cumaric acid was dissolved in water, and cumaric C10-ester was dissolved in oil to have a final concentration in the emulsion of 0.05 mM and 0.5 mM, respectively. The formation of an emulsion was assessed by observation at a magnification of 10× through an optical microscope (MT9000, Meiji Techno Co Ltd., Miyoshi, Japan) equipped with a 3-megapixel CMOS camera (Invenio 3S, DeltaPix, Smorum, Denmark). Droplets size distribution of emulsions was determined by image analysis using Image-Pro Plus 5.1 software (Media Cybernetics, Inc., Rockville, MD, USA). Three pictures for each sample were analyzed, and at least 6000 droplets were measured automatically after applying to the pictures the Hi-Gauss filter. After the preparation, emulsions were stored in tightly closed vials at room temperature and protected from light.

### 2.4. Oil-to-Water Partition Coefficient Determination

The oil-to-water partition coefficient (log P) was determined through the shake-flask method by measuring the relative concentration of the compound in the oil and water phase after partition according to Equation (1) [23]:

(1) $$log\;P=log_{10\;}\frac{{\left[compound\right]}_{oil}}{\;{\left[compound\right]}_{water}\;}\;$$

Firstly, 10 mg of the cumaric acid were solubilized in 10 mL of ultrapure water (pH 6.4, resistivity > 18 MΩ·cm) or 10 mg of the cumaric C10-ester derivative were solubilized in 10 mL of the oil (Nigella or EVO oil). Then, the obtained solution was placed in a separation funnel, and 10 mL of the other phase (oil for the acid or ultrapure water for ester) were added. The mixture was shacked for 5 min, and the separation funnel was set upright to allow the complete separation of the phases at room temperature. After 24 h, the two phases were collected and analyzed spectrophotometrically (UV-1800 spectrophotometer, Shimadzu, Kyoto, Japan) after dilution in ethanol. The concentration of the compounds was determined by building up a calibration curve in ethanol at the wavelength of 286 nm for cumaric acid and at 273 nm for cumaric C10-ester, as experimentally determined. The analyses were performed in triplicate. The acid value of the oils (mg of KOH required for the neutralization of 1 g of oil) was determined by titrating 0.2 g of the oil dissolved in 10 mL of ethanol, using a KOH standard solution (0.02 M) and phenolphthalein as pH indicator.

### 2.5. Antioxidant Assays

#### 2.5.1. Measurement of Hydrogen Peroxide/Luminol-Derived Chemiluminescence (CL)

The hydrogen peroxide (H_2_O_2_) scavenger capacity of the tested compounds was measured by a chemiluminescent assay using an Autolumat LB953 multi-tube luminometer (Berthold Co, Wildbard, Germany), according to Bordoni et al. [16]. The reaction mixture was composed of different concentrations of the tested compounds (from 0.5 to 5 mM) and 0.01 mM of luminol in Tris-HCl buffer (20 mM, pH 7.4). The reaction was followed after injection of H_2_O_2_ at a final concentration of 50 mM. The H_2_O_2_ scavenging activity of the tested compounds was measured in terms of inhibition of luminol-derived CL. Data were expressed as percent (%) inhibition of CL reaction with respect to the blank solution (luminol and H_2_O_2_ in Tris-HCl buffer or blank emulsion) and calculated as follows (Equation (2)):

(2) $$\%\;inhibition=100-\left(\frac{Sample\;CL\;area}{Blank\;CL\;area}\times 100\right)$$

Data are from three independent experiments in triple replications.

#### 2.5.2. Measurement of Superoxide/Lucigenin-Derived CL

CL measurements were performed using lucigenin as a chemiluminogenic probe to detect the superoxide radical produced by the xanthine/xanthine oxidase system. The CL was measured using an Autolumat LB953 multi-tube luminometer (Berthold Co., Wildbard, Germany) in a reaction mixture containing 0.1 U/mL xanthine oxidase, 0.15 mM lucigenin in Tris buffer (20 mM, pH 7.4), and different concentrations (from 0.5 to 5 mM) of the tested compounds. The reaction was started by injecting xanthine at a final concentration of 0.05 mM. The superoxide scavenging activity of the tested compounds was measured in terms of inhibition of lucigenin-derived CL. Data were expressed as percent (%) inhibition of CL reaction with respect to the blank solution (luminol and H_2_O_2_ in Tris-HCl buffer or blank emulsion) and calculated as reported in Equation (2). Data are from three independent experiments in triple replications.

#### 2.5.3. DPPP Assay

The capacity of cumaric acid at a concentration of 0.05 mM and C10-ester derivative of cumaric acid at a concentration of 0.5 mM to inhibit lipid hydroperoxide formation in EVO and Nigella oils, according to CL assays, was evaluated using diphenyl−1-pyrenylphosphine (DPPP). DPPP reacts with hydroperoxides and becomes highly fluorescent when oxidized. Samples were incubated with 0.001 mM DPPP in 20 mM Tris buffer pH 7.4 for 5 min in the dark. The fluorescence intensity of the samples was measured at 380 nm after excitation at 351 nm. Results are expressed as percent (%) of inhibition of lipid hydroperoxide formation reaction with respect to that of pure oils or blank emulsions and calculated as reported in Equation (3).

(3) $$\%\;inhibition=100-\left(\frac{Sample\;fluorescence\;intensity}{Blank\;fluorescence\;intensity}\times 100\right)\;$$

Data are from three independent experiments in triple replications.

#### 2.5.4. Statistical Analysis

Data are expressed as mean values ±SD. Each experiment was performed in triplicate and repeated three times. Statistical analysis was carried out using one-way analysis of variance followed by Student’s t Newman–Keuls test. A *p*-value < 0.05 was considered statistically significant.

## 3. Results

### 3.1. Synthesis and Purification of C10-Ester Derivatives of Cinnamic, Cumaric, and Ferulic Acids

**Figure 1:**
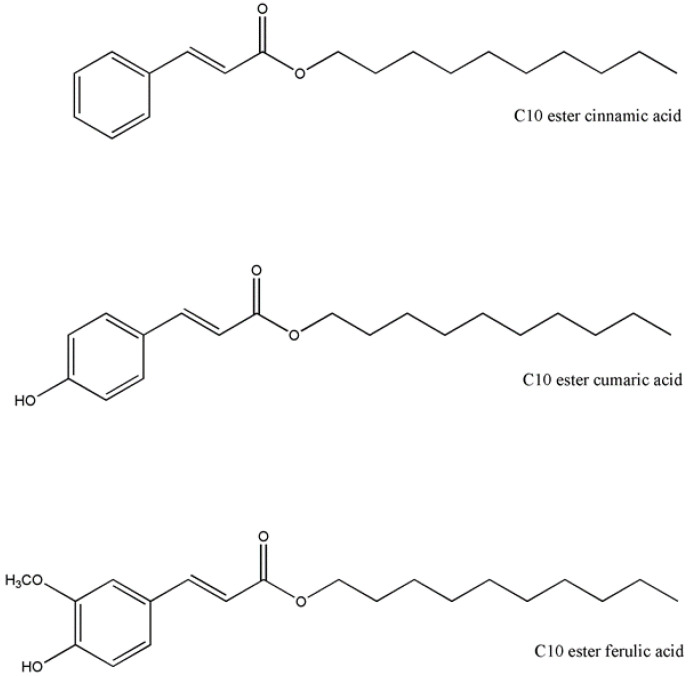
Chemical structures of the synthesized C10 ester derivatives of cinnamic, cumaric, and ferulic acids.

The hydrophobic derivatives (C10 esters) of cinnamic and hydroxycinnamic acid (cumaric and ferulic) were obtained in a one-step synthetic approach by esterification of the carboxylic group with 1-decanol using *p*-toluenesulfonic acid as a catalyst with a high yield (≥80%). The structure of the synthesized ester derivatives of ferulic acid, cinnamic acid, and cumaric acid is reported in Figure 1.

(E)-Decyl cinnamate (cinnamic C10-ester)

^1^H NMR (600 MHz, CDCl3) δ 0.891–0.918 (m,3H); 1.301–1.604 (m, 14H); 2.311–2.473 (m,2H); 4.212–4.239 (m,2H); 6.453–6.485 (m,1H); 7.401–7.415 (m,2H); 7.544–7.549 (m,2H); 7.692–7.724 (m,2H).

(E)-decyl 3-(4-hydroxyphenyl)acrylate (cumaric C10-ester)

^1^H NMR (600 MHz, CDCl3) δ 0.885–0.913 (m,3H); 1.237–1.296 (m, 14H); 1.636–1.665 (m,2H); 2.471–2.474 (m,2H); 4.029–4.055 (m,2H); 6.997–7.042 (m,1H); 7.357–7.373 (m,2H); 7.804–7.821 (m,2H).

(E)-decyl 3-(4-hydroxy-3-methoxyphenyl)acrylate (ferulic C10-ester)

^1^H NMR (600 MHz, CDCl3) δ 0.885–0.913 (m,3H); 1.232–1.289 (m, 12H); 1,297–1.303 (m,2H); 1.623–1.665 (m,2H); 2.473 (s,3H); 4.027–4.053 (m,2H); 7.285 (s,1H); 7.358–7.374 (m,2H); 7.806–7.822 (m,2H).

### 3.2. Antioxidant Properties of C10 Derivatives of Cinnamic, Cumaric, and Ferulic Acid

**Figure 2:**
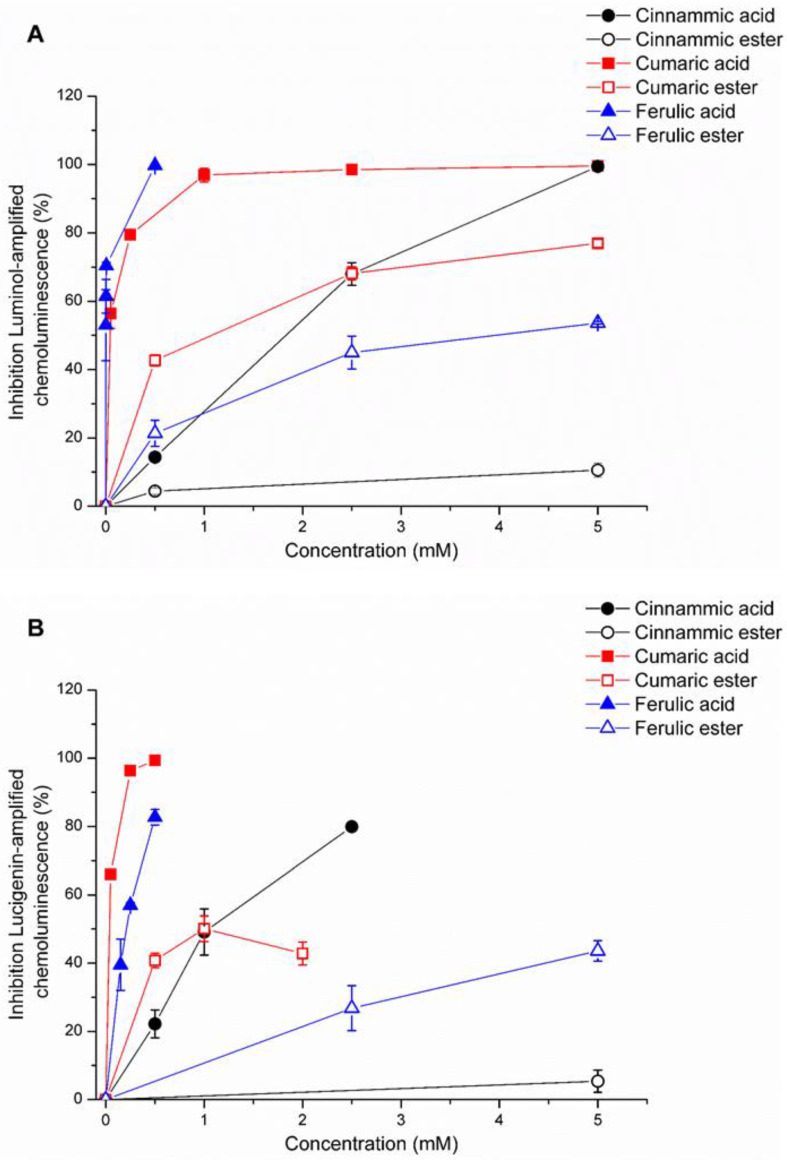
Luminol-amplified chemiluminescence (hydrogen peroxide) (**A**) and lucigenin-amplified chemiluminescence (superoxide radical) (**B**) scavenger activity of ferulic, cinnamic, and cumaric acids and their C10-esters. Data are reported as the mM of compounds (acid/ester) able to inhibit the chemiluminescence reaction. Data obtained from 3 independent experiments in 3 replicates.

Hydrogen peroxide and superoxide radical scavenger activity were measured by luminol–and lucigenin-amplified CL. The compounds showed a different capacity to neutralize reactive oxygen species (i.e., superoxide anion) and reactive molecules (i.e., hydrogen peroxide). The hydrogen peroxide antioxidant capacity, expressed as % of inhibition of luminol-amplified-CL decreases as follows: ferulic acid > cumaric acid > cumaric C10-ester > cinnamic acid > ferulic C10-ester > cinnamic C10-ester (Figure 2A). Figure 2B shows the % of inhibition of lucigenin-amplified-CL; superoxide antioxidant ability declined starting from cumaric acid > ferulic acid > cinnamic acid >cumaric C10-ester > ferulic C10-ester > cinnamic C10-ester (Figure 2B). The scavenging activity was higher for ferulic, cumaric, and cinnamic acid in comparison to their C10 ester derivatives toward both hydrogen peroxide and superoxide radical. Cumaric C10-ester displayed the highest ability to scavenger both superoxide anion and hydrogen peroxides, and it was selected for further studies together with its acid as a comparison.

According to the outcomes in Figure 2, cumaric C10-ester and cumaric acid (for reference) have also been studied for their capacity to contrast lipid hydroperoxides in EVO and *Nigella* oil. To this aim, 0.05 mM cumaric acid and 0.5 mM cumaric C10-ester were incubated with *Nigella* and EVO oil, and percent (%) hydroperoxide accumulation was measured over time up to 7 h in comparison to pure oil as control (100%). No marked differences between the two tested compounds (C10 ester derivative and cumaric acid) were observed in the ability to decrease hydroperoxide accumulation in both oils, despite a slightly lower amount of hydroperoxides being measured in Nigella sativa oil containing the cumaric C10 ester (0.5 mM). An almost constant inhibition of hydroperoxide formation was maintained in all samples for the entire observation time (up to 7 h) (Table 1).

### 3.3. Emulsion Preparation and Characterization

**Figure 3:**
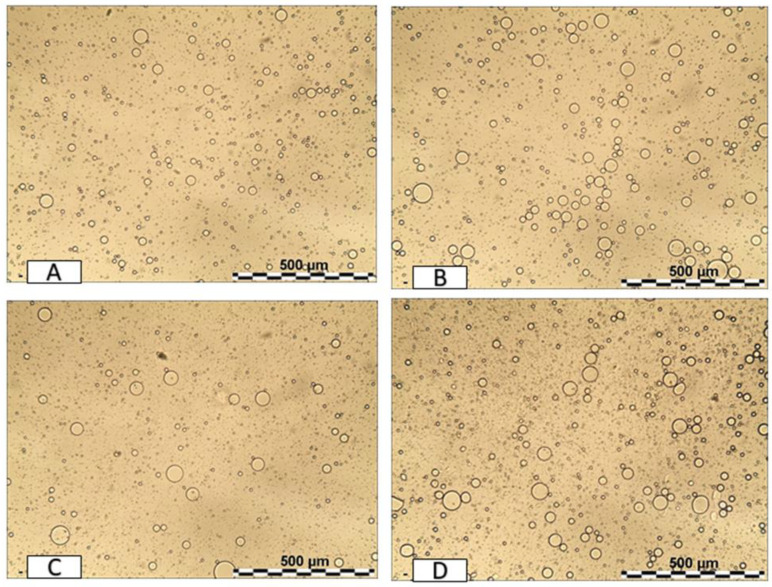
Optical microscope images (magnification 10×) for the prepared emulsions at 5% *w/w* of *Nigella* oil containing cumaric C10-ester (**A**) or cumaric acid (**B**) and for the prepared emulsions at 5% *w/w* of olive oil containing cumaric C10-ester (**C**) or cumaric acid (**D**).

Blank emulsions and emulsions containing cumaric acid or cumaric C10-ester were prepared using olive oil and *Nigella* oil at 5% *w/w* as oil phase and polysorbate 80 at 1% *w/w* as surfactant. The concentration of polysorbate 80 was set at 1% *w/w* after a preliminary screening to assess emulsion formation and stability at different percentages of surfactant (1–3% *w/w*). Emulsified systems were obtained at any tested surfactant concentrations and were stable for at least 3 months without any phase separation. A total of 1% *w/w* of polysorbate 80 was chosen as the lowest concentration of surfactant that was able to emulsify the oil phase. The optical images of blank and loaded emulsions prepared at 1% of polysorbate 80 are shown in Figure 3. The presence of oil droplets confirms the achievement of emulsified systems, and the droplet size of blank and loaded emulsions was calculated by image analysis of the pictures collected by optical microscopy.

**Figure 4:**
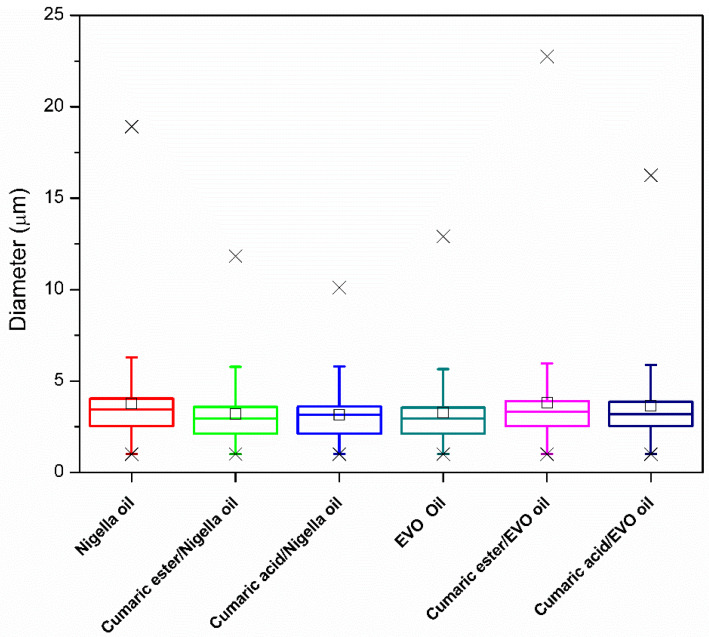
Box plot of the droplet size distributions for the prepared *Nigella* and EVO-based blank and loaded emulsions.

The droplet size results are presented using a box chart in Figure 4. In this plot, the upper and lower box horizontal borders represent the 25th and 75th percentile, the horizontal line inside the box represents the median value, while the whiskers the 5th and 95th percentile and symbols (✕) the 1st and 99th percentile of the droplet size distribution. The calculated mean droplet size is indicated by the symbol □. All emulsions, both blank and loaded ones, have a similar particle size distribution with mean values slightly higher than median values (positively skewed distribution) in the range 3–4 µm.

### 3.4. Distribution Coefficient Determination of Cumaric Acid and Its C10 Ester Derivative in EVO Oil and Nigella Oil

The oil-to-water distribution coefficients were determined to investigate whether the C10 esterification can affect the partition of the synthesized cumaric derivative between ultrapure water and the oily phase (EVO oil or *Nigella* oil), which was employed for the preparation of the emulsified systems. The determination of this chemical-physical parameter is useful to make a prediction on which phase the acid or the corresponding ester is located inside the emulsion (water or oil). Indeed, the esterification makes the cumaric derivative practically insoluble in water; therefore, the ester is almost all distributed in the oily phase, as resulted from the calculated LogP values much larger than 1 (Table 2). For cumaric C10 ester, a specific value for LogP cannot be calculated since the amount distributed in water was below the detection limit of the instrument (0.03 µg/mL). On the other side, cumaric acid has a calculated positive LogP value in the case of ultrapure water/*Nigella* oil mixture (LogP 0.136 ± 0.008, Table 2), while a calculated negative LogP value in the case of ultrapure water/EVO oil mixture (LogP −0.194 ± 0.024, Table 2). As a consequence, cumaric acid is more distributed in the oil phase in the case of the ultrapure water/*Nigella* oil mixture, while it is more distributed in ultrapure water in the case of ultrapure water/EVO oil mixture. The different extent of cumaric acid distribution in ultrapure water and oily phase can be affected by the acidity of EVO oil and *Nigella* oil, which in turn influences the ionization of the compound. As already reported [24], *Nigella* Oil is more acid than EVO oil, as resulted from the calculated acid values, which were in the range 3–4 mg/g for EVO oil and 22–23 mg/g for *Nigella* oil.

### 3.5. Antioxidant Activity of Cumaric Acid and Cumaric C10-Ester in EVO and Nigella-Based Emulsions

**Figure 5:**
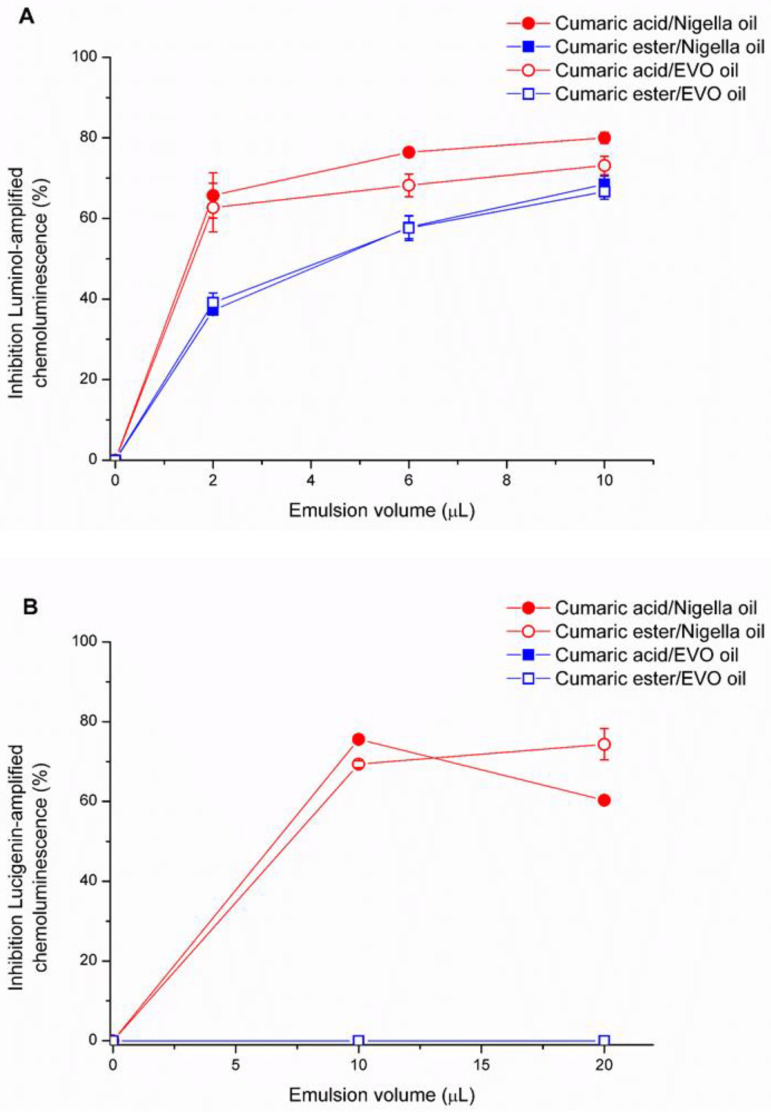
Luminol-amplified chemiluminescence (hydrogen peroxide) (**A**) and lucigenin-amplified chemiluminescence (superoxide radical) (**B**) scavenger activity of cumaric acid and C10-ester of cumaric acid in emulsions. Data are reported as µL of emulsion able to inhibit 50% of CL reaction. Data obtained from 3 independent experiments in 3 replicates.

The antioxidant activity of C10-ester derivative and cumaric acid (as reference) was studied in EVO and *Nigella*-based emulsions. Data in Figure 5 show that cumaric acid and its ester can exert a higher scavenger activity toward hydrogen peroxide (Figure 5A) and superoxide anion (Figure 5B) in *Nigella* oil emulsion than in EVO emulsions. Cumaric acid and cumaric C-10 ester in EVO emulsion display an antioxidant activity against hydrogen peroxide-CL signal (Figure 5A), while they do not show any antioxidant capacity toward superoxide radical (Figure 5B).

Hydroperoxide formation was measure also in the emulsions; the lipid hydroperoxides were reduced (about 20%) during the 7 h of incubation only in the sample containing the cumaric ester in the emulsion prepared with *Nigella sativa* oil.

## 4. Discussion

Cinnamic and *p*-hydroxycinnamic acids are phenolic compounds belonging to the class of phenylpropanoids and are the main constituents of the cell wall of many plants [25]. Although they are natural products found abundantly in fruits, vegetables, cereals, and seeds, many synthetic derivatives have been prepared by esterification of the -COOH lateral groups with alcohols of a different chain length with the aim to increase the lipophilicity of the compounds and, therefore, their partitioning in lipid membranes, including natural body barriers as the skin [26]. The antioxidant and anti-inflammatory activity of several hydroxycinnamic derivatives (e.g., ferulic acid, rosmarinic acid), mainly esterified with a short alcohol (methyl, ethyl, and butyl derivatives), have been investigated [27,28]. All these compounds are reported to maintain the antioxidant activity since this modification does not regard the -OH group, which is directly involved in the scavenging mechanism [1]. However, the relationship between the elongation grade of the acyl chain length and the potency of the antioxidant effect of these derivatives has never been definitely stated due to the different experimental conditions applied and the scarce water solubility of the homologs with a longer chain. At the same time, poor information is available in the literature regarding the direct comparison of different hydroxycinnamic acid esterified with the same alcohol [29]. Therefore, in the present work, the antioxidant activity of three esters prepared from cinnamic, cumaric, and ferulic acid and a medium-chain C10-alcohol was investigated. As already reported [30], ferulic acid and cumaric acid have a more pronounced scavenger activity than cinnamic acid. However, when tested in an aqueous environment, all acids showed a marked higher scavenging capacity both toward superoxide radical and hydrogen peroxide than the corresponding C10 esters. The lower activity of C10-esters could be explained by the complex mesomeric/inductive effects exerted by the ethylene groups of the lateral chains on the stability of the semiquinone intermediate, forming during the scavenging process [31]. Then, the most performing derivative (C10 cumaric esters) was selected to be incorporated inside a model emulsified system prepared using polysorbate 80 as surfactant and two biologically active oils (*Nigella* and EVO) as oily phase. The obtained results have confirmed the remarkable superoxide anion capacity of *Nigella sativa* oil compared with EVO as observed in a previous study, where *Nigella sativa* oil showed a stronger effect than EVO in the gene suppression of pro-inflammatory cytokines (i.e., IL-1β, and MCP1) [17]. Although, literature is rich in studies that demonstrate the key antioxidant capacity of *Nigella sativa* oil, related to the high content of thymoquinone, in animals and humans at different experimental conditions [16,32,33], the association of this oil in formulations with cinnamic and hydroxycinnamic acids results to be a novelty within the field of antioxidants. These emulsified systems containing 5% *w/w* of oil and 1% *w/w* of polysorbate 80 can be considered as prototypes for developing formulations with antioxidant activity that can be applied topically on the skin or mucosa. These formulations can combine the beneficial effects of the two oils with the antioxidant activity of the synthesized compound, which, being lipophilic, can be simply incorporated into the formulations by direct solubilization into the oily phase.

## 5. Conclusions

In conclusion, the present study shows a higher scavenger activity of cinnamic and hydroxycinnamic acids than the corresponding C10 esters toward superoxide radicals and hydrogen peroxide. As regards the acids, ferulic acid and cumaric acid demonstrate to be more active with respect to cinnamic acid as antioxidants. Cumaric acid and its C10 ester derivative were selected to prepare emulsions containing *Nigella sativa* or EVO oils as oily phase. *Nigella sativa* oil-based emulsions loaded with cumaric acid or its C10-ester demonstrate a higher capacity than EVO to scavenger superoxide anion and hydrogen peroxide. Moreover, cumaric acid and C10-ester demonstrate to be active against hydroperoxide formation (up to 7 h) in both tested oils, while only cumaric C10 ester was active in *Nigella sativa* oil-based emulsion. Further studies are required to evaluate the long-term activity of these compounds for the development of new emulsion formulations in combination with *Nigella Sativa* oil.

## Figures and Tables

**Table 1:** Cumaric acid and cumaric C10-ester ability to protect hydroperoxide formation in *Nigella sativa* oil and extra-virgin olive (EVO) oil. Inhibition of percent (%) hydroperoxide accumulation in the samples was calculated vs. control (pure oils), set as 100%. Incubation was carried out in the dark and in the absence of oxygen. Data obtained from 3 independent experiments in 3 replicates, *p* > 0.05.

	Inhibition (%) of Hydroperoxide Accumulation			
	*Nigella* Oil		EVO Oil	
Incubation Time (h)	Cumaric Acid	Cumaric C10-Ester	Cumaric Acid	Cumaric C10-Ester
0	84.4 ± 2.72	73.4 ± 2.14	84.6 ± 18.80	85.6 ± 10.05
1	82.4 ± 3.45	66.2 ± 2.32	69.3 ± 17.55	86.3 ± 3.24
2	83.5 ± 8.76	64.7 ± 17.92	96.7 ± 0.81	98.3 ± 1.99
3	77.1 ± 16.31	73.8 ± 10.31	83.4 ± 9.87	85.8 ± 7.04
4	73.7 ± 11.55	74.3 ± 10.65	83.0 ± 12.64	90.8 ± 21.30
5	81.4 ± 2.13	70.2 ± 13.71	83.8 ± 6.70	88.8 ± 25.75
6	77.1 ± 16.79	73.1 ± 24.71	89.4 ± 4.88	82.9 ± 7.35
7	93.2 ± 13.61	75.7 ± 5.70	92.6 ± 7.54	88.7 ± 9.31

**Table 2:** Calculated percentages (%) and oil-to-water distribution coefficients (LogP) of cumaric acid and cumaric C10-ester in the ultrapure water/EVO oil and ultrapure water/*Nigella* oil mixtures.

	EVO Oil			*Nigella* Oil		
	% of Total Compound in Oil Phase	% of Total Compound in Water Phase	Log P	% of Total Compoundin Oil Phase	% of Total Compound in Water Phase	Log P
**Cumaric Acid**	39.01 ± 1.33	60.99 ± 1.33	−0.194 ± 0.024	57.77 ± 0.45	42.23 ± 0.45	0.136 ± 0.008
**Cumaric C10-Ester**	>99.99	˂0.01	>2.76	>99.99	˂0.01	>2.76

## Data Availability

The data is contained within the article.
